# *N*-[4-(4-Chloro­benzene­sulfonamido)­phenyl­sulfon­yl]acetamide

**DOI:** 10.1107/S1600536812023434

**Published:** 2012-05-26

**Authors:** Islam Ullah Khan, Ejaz, Sidra Farid, William T. A. Harrison

**Affiliations:** a Materials Chemistry Laboratry, Department of Chemistry, GC University, Lahore 54000, Pakistan; b Department of Chemistry, University of Aberdeen, Meston Walk, Aberdeen AB24 3UE, Scotland

## Abstract

In the title compound, C_14_H_13_ClN_2_O_5_S_2_, the dihedral angles between the central benzene ring and the pendant chloro­benzene ring and the *N*-acetyl group are 82.35 (5) and 79.71 (6)°, respectively, and the overall conformation of the mol­ecule approximates to a U shape. Both the C—S—N—C conformations are *gauche*, but with opposite senses [torsion angles = −59.29 (15) and 63.68 (16)°]. An intra­molecular C—H⋯O inter­action generates an *S*(6) ring. In the crystal, inversion dimers linked by pairs of N—H⋯O hydrogen bonds generate *R*_2_^2^(20) loops. A second N—H⋯O hydrogen bond links the dimers into (101) layers.

## Related literature

For related structures, see: Ashfaq *et al.* (2009 ▶, 2010 ▶).
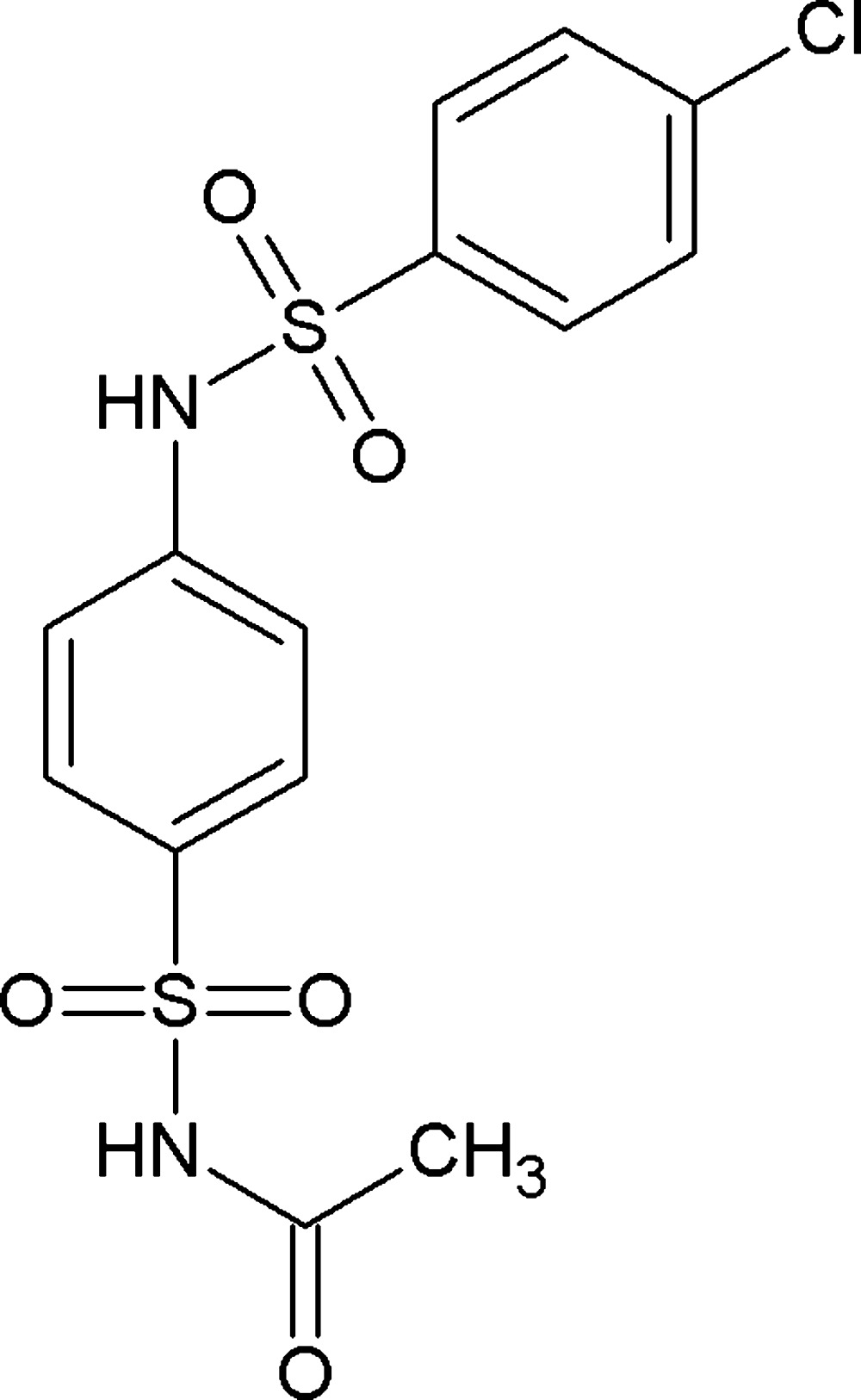


## Experimental

### 

#### Crystal data

C_14_H_13_ClN_2_O_5_S_2_
*M* *_r_* = 388.83
Monoclinic, 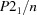

*a* = 9.7452 (2) Å
*b* = 9.9905 (2) Å
*c* = 17.3968 (3) Å
β = 99.870 (1)°
*V* = 1668.67 (6) Å^3^
*Z* = 4
Mo *K*α radiation
μ = 0.51 mm^−1^
*T* = 296 K
0.40 × 0.20 × 0.10 mm

#### Data collection

Bruker APEXII CCD diffractometer
Absorption correction: multi-scan (*SADABS*; Bruker, 2007 ▶) *T*_min_ = 0.823, *T*_max_ = 0.951
15966 measured reflections
4146 independent reflections
3267 reflections with *I* > 2σ(*I*)
*R*_int_ = 0.026

#### Refinement

*R*[*F* ^2^ > 2σ(*F* ^2^)] = 0.034
*wR*(*F* ^2^) = 0.092
*S* = 1.03
4146 reflections
226 parameters
H atoms treated by a mixture of independent and constrained refinement
Δρ_max_ = 0.30 e Å^−3^
Δρ_min_ = −0.37 e Å^−3^

### 

Data collection: *APEX2* (Bruker, 2007 ▶); cell refinement: *SAINT* (Bruker, 2007 ▶); data reduction: *SAINT*; program(s) used to solve structure: *SHELXS97* (Sheldrick, 2008 ▶); program(s) used to refine structure: *SHELXL97* (Sheldrick, 2008 ▶); molecular graphics: *ORTEP-3* (Farrugia, 1997 ▶); software used to prepare material for publication: *SHELXL97*.

## Supplementary Material

Crystal structure: contains datablock(s) I, global. DOI: 10.1107/S1600536812023434/su2434sup1.cif

Structure factors: contains datablock(s) I. DOI: 10.1107/S1600536812023434/su2434Isup2.hkl

Supplementary material file. DOI: 10.1107/S1600536812023434/su2434Isup3.cml

Additional supplementary materials:  crystallographic information; 3D view; checkCIF report

## Figures and Tables

**Table 1:** Hydrogen-bond geometry (Å, °)

*D*—H⋯*A*	*D*—H	H⋯*A*	*D*⋯*A*	*D*—H⋯*A*
N1—H1⋯O5^i^	0.85 (2)	1.97 (2)	2.8070 (19)	166 (2)
N2—H2⋯O1^ii^	0.87 (2)	2.10 (2)	2.9510 (19)	166.3 (19)
C12—H12⋯O2	0.93	2.44	3.074 (2)	126

Symmetry codes: (i) 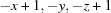; (ii) 
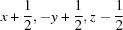
.

**Fig. 1.**
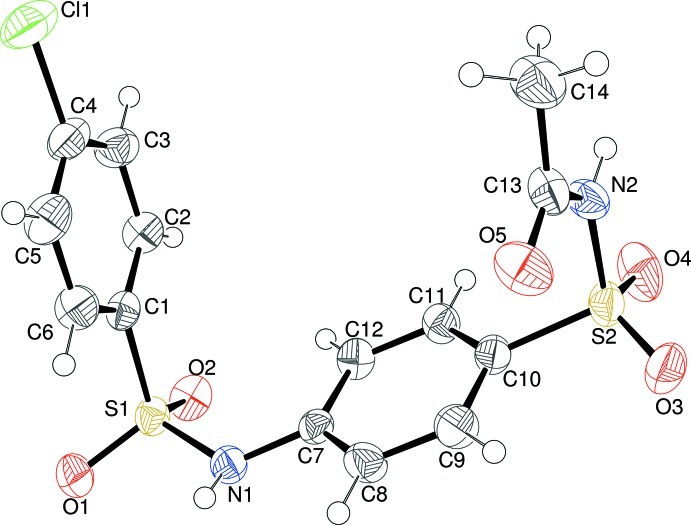
The molecular structure of (I) showing 50% displacement ellipsoids.

**Fig. 2.**
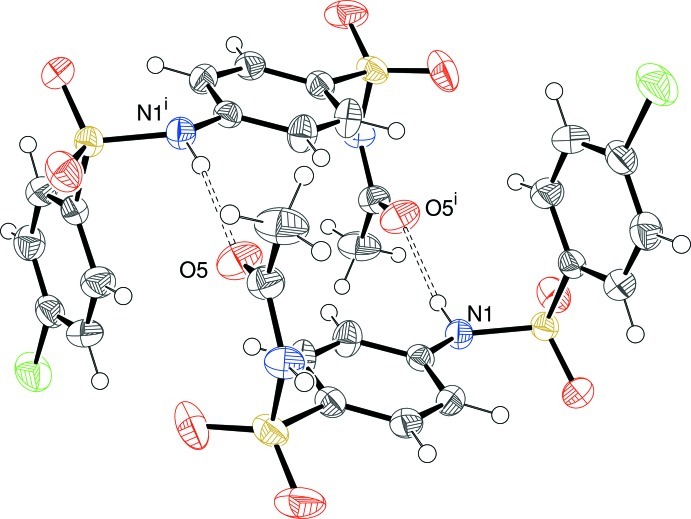
An inversion dimer in the crystal of (I), which generates an R22(20) loop. Symmetry code: (i) 1–x, –y, 1–z.

## supplementary crystallographic information

### Comment

As part of our ongoing structural studies of sulfonamides (Ashfaq *et al.*, 2009, 2010), the synthesis and structure of the title compound, (I), (Fig. 1), are now described.

The dihedral angle between the central (C7—C12) benzene ring and the pendant (C1—C6) chlorobenzene ring is 82.35 (50)°. The dihedral angle between the central ring and the C13/C14/N2/O5 amide fragment is 79.71 (60)°, and overall, the molecule adpots an approximate U shape. The conformation of the C1—S1—N1—C7 fragment is *gauche* [-59.29 (15)°], and the torsion angle for C10—S2—N2—C13 of 63.68 (16)° indicates the same thing, but in an opposite sense. The bond-angle sums for N1 and N2 are 351.4 and 360.0°, respectively. An intramolecular C—H···O interaction (Table 1) generates an S(6) ring.

In the crystal, inversion dimers linked by pairs of N—H···O hydrogen bonds generate *R*_2_^2^(20) loops (Fig. 2). The other N—H···O hydrogen bonds links the dimers into (101) layers. Centrosymmetric *R*_2_^2^(20) loops were also observed in the crystal structures of the related compounds *N*-acetyl-4-(benzenesulfonamido)-benzenesulfonamide (Ashfaq *et al.*, 2009) and *N*-[4-(*p*-toluenesulfonamido)phenylsulfonyl]acetamide (Ashfaq *et al.*, 2010).

### Experimental

Sodium sulfacetamide (0.236 g, 1.0 mmol) was dissolved in 30 ml distllled water in a 100-ml round bottom flask and the pH was adjusted to 8.0 using Na_2_CO_3_ solution (3%). 4-Chlorobenzenesulfonyl chloride (0.422 g, 2.0 mmol) was added and the mixture was stirred at 50 °C for about 5 h. The pH was adjusted to 3.0 using HCl (3 N) and the resulting white precipitate was filtered, washed and dried. Colourless blocks of (I) were recrystallized from methanol solution at room temperature. This compound has been deposited to CSD with CCDC No. 859957.

### Refinement

The N-bound H atoms were located in a difference map and their positions and *U*_iso_ values were freely refined. The C-bound hydrogen atoms were placed in calculated positions (C—H = 0.93–0.96 Å) and refined as riding atoms with *U*_iso_(H) = 1.2*U*_eq_(C) or 1.5*U*_eq_(methyl C).

### Crystal data

**Table:**

C_14_H_13_ClN_2_O_5_S_2_	*F*(000) = 800
*M**_r_* = 388.83	*D*_x_ = 1.548 Mg m^−^^3^
Monoclinic, *P*2_1_/*n*	Mo *K*α radiation, λ = 0.71073 Å
Hall symbol: -P 2yn	Cell parameters from 5590 reflections
*a* = 9.7452 (2) Å	θ = 2.4–28.1°
*b* = 9.9905 (2) Å	µ = 0.51 mm^−^^1^
*c* = 17.3968 (3) Å	*T* = 296 K
β = 99.870 (1)°	Block, colourless
*V* = 1668.67 (6) Å^3^	0.40 × 0.20 × 0.10 mm
*Z* = 4	

### Data collection

**Table:**

Bruker APEXII CCD diffractometer	4146 independent reflections
Radiation source: fine-focus sealed tube	3267 reflections with *I* > 2σ(*I*)
Graphite monochromator	*R*_int_ = 0.026
ω scans	θ_max_ = 28.3°, θ_min_ = 2.9°
Absorption correction: multi-scan (*SADABS*; Bruker, 2007)	*h* = −12→12
*T*_min_ = 0.823, *T*_max_ = 0.951	*k* = −13→13
15966 measured reflections	*l* = −21→23

### Refinement

**Table:**

Refinement on *F*^2^	Primary atom site location: structure-invariant direct methods
Least-squares matrix: full	Secondary atom site location: difference Fourier map
*R*[*F*^2^ > 2σ(*F*^2^)] = 0.034	Hydrogen site location: difmap (N-H) and geom (C-H)
*wR*(*F*^2^) = 0.092	H atoms treated by a mixture of independent and constrained refinement
*S* = 1.03	*w* = 1/[σ^2^(*F*_o_^2^) + (0.0429*P*)^2^ + 0.5555*P*] where *P* = (*F*_o_^2^ + 2*F*_c_^2^)/3
4146 reflections	(Δ/σ)_max_ = 0.001
226 parameters	Δρ_max_ = 0.30 e Å^−^^3^
0 restraints	Δρ_min_ = −0.37 e Å^−^^3^

### Special details

**Table:**

Geometry. All e.s.d.'s (except the e.s.d. in the dihedral angle between two l.s. planes) are estimated using the full covariance matrix. The cell e.s.d.'s are taken into account individually in the estimation of e.s.d.'s in distances, angles and torsion angles; correlations between e.s.d.'s in cell parameters are only used when they are defined by crystal symmetry. An approximate (isotropic) treatment of cell e.s.d.'s is used for estimating e.s.d.'s involving l.s. planes.
Refinement. Refinement of *F*^2^ against ALL reflections. The weighted *R*-factor *wR* and goodness of fit *S* are based on *F*^2^, conventional *R*-factors *R* are based on *F*, with *F* set to zero for negative *F*^2^. The threshold expression of *F*^2^ > σ(*F*^2^) is used only for calculating *R*-factors(gt) *etc*. and is not relevant to the choice of reflections for refinement. *R*-factors based on *F*^2^ are statistically about twice as large as those based on *F*, and *R*- factors based on ALL data will be even larger.

### Fractional atomic coordinates and isotropic or equivalent isotropic displacement parameters (Å^2^)

**Table:**

	*x*	*y*	*z*	*U*_iso_*/*U*_eq_	
C1	0.20974 (17)	0.33396 (16)	0.55613 (9)	0.0306 (3)	
C2	0.18774 (18)	0.44608 (18)	0.50941 (10)	0.0368 (4)	
H2A	0.1078	0.4530	0.4718	0.044*	
C3	0.2851 (2)	0.54835 (18)	0.51874 (11)	0.0415 (4)	
H3	0.2711	0.6248	0.4879	0.050*	
C4	0.40247 (19)	0.53498 (18)	0.57432 (11)	0.0402 (4)	
C5	0.4280 (2)	0.4213 (2)	0.62017 (11)	0.0449 (5)	
H5	0.5095	0.4135	0.6566	0.054*	
C6	0.33066 (19)	0.31992 (19)	0.61088 (10)	0.0393 (4)	
H6	0.3458	0.2427	0.6410	0.047*	
C7	0.21126 (16)	0.06282 (15)	0.44896 (9)	0.0274 (3)	
C8	0.30854 (19)	−0.03793 (17)	0.44559 (10)	0.0372 (4)	
H8	0.3333	−0.0950	0.4879	0.045*	
C9	0.3684 (2)	−0.05378 (18)	0.38001 (11)	0.0404 (4)	
H9	0.4333	−0.1213	0.3779	0.048*	
C10	0.33119 (17)	0.03193 (16)	0.31703 (9)	0.0315 (3)	
C11	0.23330 (18)	0.13100 (17)	0.31958 (10)	0.0335 (4)	
H11	0.2084	0.1875	0.2770	0.040*	
C12	0.17231 (17)	0.14656 (17)	0.38490 (9)	0.0332 (4)	
H12	0.1055	0.2126	0.3862	0.040*	
C13	0.65205 (18)	0.12228 (19)	0.30551 (10)	0.0363 (4)	
C14	0.7580 (2)	0.2294 (2)	0.30576 (12)	0.0545 (5)	
H14A	0.7909	0.2578	0.3584	0.082*	
H14B	0.7170	0.3041	0.2755	0.082*	
H14C	0.8347	0.1955	0.2835	0.082*	
N1	0.15644 (15)	0.07329 (14)	0.51825 (8)	0.0311 (3)	
H1	0.202 (2)	0.029 (2)	0.5560 (13)	0.050 (6)*	
N2	0.53926 (15)	0.12827 (16)	0.24656 (8)	0.0345 (3)	
H2	0.531 (2)	0.191 (2)	0.2111 (12)	0.046 (6)*	
O1	0.06025 (14)	0.17153 (13)	0.62438 (7)	0.0432 (3)	
O2	−0.03065 (12)	0.24552 (13)	0.48970 (7)	0.0414 (3)	
O3	0.46830 (16)	−0.11310 (14)	0.23297 (9)	0.0538 (4)	
O4	0.31838 (14)	0.06823 (17)	0.16787 (7)	0.0557 (4)	
O5	0.66166 (14)	0.03331 (15)	0.35379 (8)	0.0496 (3)	
S1	0.08358 (4)	0.20587 (4)	0.54746 (2)	0.03105 (11)	
S2	0.41012 (5)	0.01756 (5)	0.23429 (2)	0.03680 (12)	
Cl1	0.52276 (6)	0.66492 (5)	0.58794 (4)	0.06056 (17)	

### Atomic displacement parameters (Å^2^)

**Table:**

	*U*^11^	*U*^22^	*U*^33^	*U*^12^	*U*^13^	*U*^23^
C1	0.0309 (8)	0.0310 (8)	0.0301 (8)	0.0009 (6)	0.0061 (7)	−0.0060 (6)
C2	0.0343 (9)	0.0359 (9)	0.0386 (9)	0.0022 (7)	0.0018 (7)	−0.0008 (7)
C3	0.0452 (10)	0.0318 (9)	0.0481 (11)	0.0008 (8)	0.0099 (8)	0.0002 (8)
C4	0.0373 (10)	0.0349 (9)	0.0499 (11)	−0.0063 (7)	0.0119 (8)	−0.0139 (8)
C5	0.0354 (10)	0.0495 (11)	0.0452 (11)	−0.0022 (8)	−0.0062 (8)	−0.0064 (8)
C6	0.0397 (10)	0.0391 (9)	0.0368 (9)	0.0019 (8)	−0.0002 (8)	0.0023 (7)
C7	0.0270 (8)	0.0265 (7)	0.0285 (8)	−0.0034 (6)	0.0041 (6)	−0.0032 (6)
C8	0.0440 (10)	0.0324 (9)	0.0369 (9)	0.0080 (8)	0.0118 (8)	0.0081 (7)
C9	0.0444 (10)	0.0335 (9)	0.0462 (10)	0.0103 (8)	0.0160 (8)	0.0034 (7)
C10	0.0316 (8)	0.0331 (8)	0.0305 (8)	−0.0031 (7)	0.0075 (7)	−0.0047 (6)
C11	0.0358 (9)	0.0357 (9)	0.0276 (8)	0.0016 (7)	0.0013 (7)	0.0011 (7)
C12	0.0319 (9)	0.0347 (9)	0.0322 (8)	0.0064 (7)	0.0028 (7)	−0.0001 (7)
C13	0.0341 (9)	0.0478 (10)	0.0278 (8)	−0.0007 (8)	0.0072 (7)	−0.0003 (7)
C14	0.0464 (12)	0.0745 (15)	0.0419 (11)	−0.0209 (11)	0.0057 (9)	−0.0011 (10)
N1	0.0337 (8)	0.0299 (7)	0.0298 (7)	0.0027 (6)	0.0062 (6)	0.0004 (6)
N2	0.0376 (8)	0.0401 (8)	0.0254 (7)	−0.0053 (6)	0.0040 (6)	0.0051 (6)
O1	0.0490 (8)	0.0476 (7)	0.0375 (7)	−0.0043 (6)	0.0200 (6)	−0.0056 (6)
O2	0.0283 (6)	0.0455 (7)	0.0485 (7)	0.0045 (5)	0.0015 (5)	−0.0062 (6)
O3	0.0631 (10)	0.0419 (8)	0.0630 (9)	−0.0060 (7)	0.0300 (7)	−0.0204 (7)
O4	0.0457 (8)	0.0911 (12)	0.0276 (7)	−0.0079 (8)	−0.0012 (6)	−0.0068 (7)
O5	0.0469 (8)	0.0599 (9)	0.0397 (7)	0.0029 (7)	0.0007 (6)	0.0163 (6)
S1	0.0282 (2)	0.0340 (2)	0.0318 (2)	0.00023 (16)	0.00766 (16)	−0.00486 (16)
S2	0.0377 (2)	0.0439 (3)	0.0297 (2)	−0.00666 (19)	0.00829 (17)	−0.01026 (17)
Cl1	0.0523 (3)	0.0466 (3)	0.0847 (4)	−0.0174 (2)	0.0175 (3)	−0.0224 (3)

### Geometric parameters (Å, º)

**Table:**

C1—C2	1.379 (2)	C10—C11	1.381 (2)
C1—C6	1.389 (2)	C10—S2	1.7504 (16)
C1—S1	1.7630 (17)	C11—C12	1.379 (2)
C2—C3	1.385 (3)	C11—H11	0.9300
C2—H2A	0.9300	C12—H12	0.9300
C3—C4	1.372 (3)	C13—O5	1.215 (2)
C3—H3	0.9300	C13—N2	1.370 (2)
C4—C5	1.386 (3)	C13—C14	1.487 (3)
C4—Cl1	1.7380 (18)	C14—H14A	0.9600
C5—C6	1.378 (3)	C14—H14B	0.9600
C5—H5	0.9300	C14—H14C	0.9600
C6—H6	0.9300	N1—S1	1.6249 (14)
C7—C8	1.391 (2)	N1—H1	0.85 (2)
C7—C12	1.393 (2)	N2—S2	1.6615 (15)
C7—N1	1.404 (2)	N2—H2	0.87 (2)
C8—C9	1.377 (2)	O1—S1	1.4369 (13)
C8—H8	0.9300	O2—S1	1.4220 (13)
C9—C10	1.389 (2)	O3—S2	1.4249 (15)
C9—H9	0.9300	O4—S2	1.4270 (15)
			
C2—C1—C6	120.93 (16)	C10—C11—H11	119.8
C2—C1—S1	120.23 (13)	C11—C12—C7	119.66 (15)
C6—C1—S1	118.84 (13)	C11—C12—H12	120.2
C1—C2—C3	119.74 (17)	C7—C12—H12	120.2
C1—C2—H2A	120.1	O5—C13—N2	120.40 (17)
C3—C2—H2A	120.1	O5—C13—C14	123.58 (17)
C4—C3—C2	118.85 (17)	N2—C13—C14	116.01 (16)
C4—C3—H3	120.6	C13—C14—H14A	109.5
C2—C3—H3	120.6	C13—C14—H14B	109.5
C3—C4—C5	122.09 (17)	H14A—C14—H14B	109.5
C3—C4—Cl1	119.00 (15)	C13—C14—H14C	109.5
C5—C4—Cl1	118.91 (15)	H14A—C14—H14C	109.5
C6—C5—C4	118.85 (17)	H14B—C14—H14C	109.5
C6—C5—H5	120.6	C7—N1—S1	125.47 (12)
C4—C5—H5	120.6	C7—N1—H1	113.3 (15)
C5—C6—C1	119.49 (17)	S1—N1—H1	112.5 (15)
C5—C6—H6	120.3	C13—N2—S2	124.05 (13)
C1—C6—H6	120.3	C13—N2—H2	121.7 (14)
C8—C7—C12	119.68 (14)	S2—N2—H2	114.3 (14)
C8—C7—N1	116.91 (14)	O2—S1—O1	119.66 (8)
C12—C7—N1	123.41 (14)	O2—S1—N1	109.71 (8)
C9—C8—C7	120.46 (16)	O1—S1—N1	104.12 (8)
C9—C8—H8	119.8	O2—S1—C1	107.95 (8)
C7—C8—H8	119.8	O1—S1—C1	108.23 (8)
C8—C9—C10	119.48 (16)	N1—S1—C1	106.43 (7)
C8—C9—H9	120.3	O3—S2—O4	120.51 (9)
C10—C9—H9	120.3	O3—S2—N2	108.49 (8)
C11—C10—C9	120.35 (15)	O4—S2—N2	102.91 (8)
C11—C10—S2	119.25 (13)	O3—S2—C10	108.70 (8)
C9—C10—S2	120.39 (13)	O4—S2—C10	109.52 (8)
C12—C11—C10	120.35 (16)	N2—S2—C10	105.64 (8)
C12—C11—H11	119.8		
			
C6—C1—C2—C3	−2.2 (3)	C12—C7—N1—S1	−21.5 (2)
S1—C1—C2—C3	177.56 (13)	O5—C13—N2—S2	−1.1 (3)
C1—C2—C3—C4	0.5 (3)	C14—C13—N2—S2	178.90 (14)
C2—C3—C4—C5	1.4 (3)	C7—N1—S1—O2	57.26 (15)
C2—C3—C4—Cl1	−178.37 (14)	C7—N1—S1—O1	−173.53 (13)
C3—C4—C5—C6	−1.6 (3)	C7—N1—S1—C1	−59.29 (15)
Cl1—C4—C5—C6	178.15 (14)	C2—C1—S1—O2	−1.39 (16)
C4—C5—C6—C1	0.0 (3)	C6—C1—S1—O2	178.34 (13)
C2—C1—C6—C5	1.9 (3)	C2—C1—S1—O1	−132.27 (14)
S1—C1—C6—C5	−177.80 (14)	C6—C1—S1—O1	47.46 (16)
C12—C7—C8—C9	1.2 (3)	C2—C1—S1—N1	116.33 (14)
N1—C7—C8—C9	−179.22 (16)	C6—C1—S1—N1	−63.94 (15)
C7—C8—C9—C10	0.1 (3)	C13—N2—S2—O3	−52.72 (16)
C8—C9—C10—C11	−1.0 (3)	C13—N2—S2—O4	178.53 (15)
C8—C9—C10—S2	177.83 (14)	C13—N2—S2—C10	63.68 (16)
C9—C10—C11—C12	0.5 (3)	C11—C10—S2—O3	−160.62 (14)
S2—C10—C11—C12	−178.32 (13)	C9—C10—S2—O3	20.54 (17)
C10—C11—C12—C7	0.8 (3)	C11—C10—S2—O4	−27.10 (16)
C8—C7—C12—C11	−1.7 (2)	C9—C10—S2—O4	154.06 (15)
N1—C7—C12—C11	178.78 (15)	C11—C10—S2—N2	83.11 (15)
C8—C7—N1—S1	159.00 (13)	C9—C10—S2—N2	−95.73 (16)

### Hydrogen-bond geometry (Å, º)

**Table:**

*D*—H···*A*	*D*—H	H···*A*	*D*···*A*	*D*—H···*A*
N1—H1···O5^i^	0.85 (2)	1.97 (2)	2.8070 (19)	166 (2)
N2—H2···O1^ii^	0.87 (2)	2.10 (2)	2.9510 (19)	166.3 (19)
C12—H12···O2	0.93	2.44	3.074 (2)	126

Symmetry codes: (i) −*x*+1, −*y*, −*z*+1; (ii) *x*+1/2, −*y*+1/2, *z*−1/2.
